# Quantity and quality of retrograde menstruation: a case control study

**DOI:** 10.1186/1477-7827-7-123

**Published:** 2009-10-30

**Authors:** Attila Bokor, Sophie Debrock, Maria Drijkoningen, Willy Goossens, Vilmos Fülöp, Thomas D'Hooghe

**Affiliations:** 1 Leuven University Fertility Center, UZ Gasthuisberg, KULeuven, Belgium; 2 Department of Pathology, UZ Gasthuisberg, KULeuven, Belgium; 3 Department of Laboratory Medicine, KULeuven, Belgium; 4 National Health Centre, Budapest, Hungary

## Abstract

**Background:**

The purpose of this study was to test the hypothesis that menstruation is associated with a higher concentration of endometrial cells in peritoneal fluid(PF) and with increased white and red blood cell concentration in PF when compared to nonmenstrual phases of the cycle.

**Methods:**

PF was obtained at laparoscopy from 107 women with endometriosis (n = 59) and controls with a normal pelvis (n = 48) during the luteal (n = 46), follicular (n = 38) or menstrual (n = 23) phase of the cycle. Endometriosis was classified according to the classification of the American Society for Reproductive Medicine (rAFS into minimal (n = 25), mild(n = 20), moderate(n = 6) and severe(n = 8) disease. Cell counts (leucocytes, erythrocytes, thrombocytes) were determined on a cell counter. In a subset of 32 patients (13 controls and 19 women with endometriosis), PF was fixed, processed and thinlayers were prepared and stained with Papanicolaou method and with immunocytochemistry using monoclonal antibodies against cytokeratin 7(CK 7), CK 8/18, Ber-Ep4, vimentin, calretinin and CD68. Ber-Ep4 is a marker for cells with epithelial origin (in some cases for mesothelial cells as well). CD68 is specific for cells from monocyte/macrophage lineage; CK7 and CK8/18 are markers for both endometrial epithelial and mesothelial cells, whereas calretinin and vimentin are markers for both endometrial stromal and mesothelial cells.

**Results:**

In comparison with the nonmenstrual phase of the cycle, analysis of PF during menstruation showed an increased concentration of leucocytes (3.3 × 10^9^/L vs 0.8 × 10^9^/L, P = 0.03), erythrocytes (0.3 × 10^12^/L vs 0.02 × 10^12^/L, P = 0.006), hematocrit (0.03 L/L vs 0.003 L/L, P = 0.01) and hemoglobin (0.8 g/dL vs 0.1 g/dL, P = 0.01). Mesothelial cells stained positively with CK7, CK8/18, vimentin, and calretinin. Cells positive for Ber-Ep4 were not observed, except in 2 patients with endometriosis investigated during menses. In all patients 50-98% of single cells were strongly positive for both vimentin and CD68.

**Conclusion:**

When compared to nonmenstrual phases of the cycle, menstruation is associated with an increased concentration of red and white blood cells in PF. However, the presence of EM cells that are detectable by immunohistochemistry in PF is low during all phases of the cycle, including menstruation.

## Background

Endometriosis is characterized by the presence and growth of endometrial-like tissue outside the uterus and occurs in 10% of women of reproductive age. The pathogenesis of endometriosis can to a certain extent be explained by retrograde menstruation of endometrial tissue sloughed through patent fallopian tubes into the peritoneal cavity [1]. However, it has never been shown that the prevalence of endometrial (EM) cells in peritoneal fluid (PF) is higher in women with endometriosis than in controls during menstruation. In fact, the cytology of retrograde menstruation has never been studied in depth.

Based on epidemiological and experimental data, it can be hypothesized that the quantity of retrograde menstruation and the consecutively flushed endometrial cells play an important role in the development of endometriosis [2]. In previous research, retrograde menstruation, defined as red stained PF [3], has been observed during culdoscopy in 50% [4], and during laparoscopy in 70-90% of patients at the time of menstruation [5]. However, the presence of red blood cells in PF is not a proof of the presence of viable EM cells at the time of menstruation. Furthermore, in most studies the identification of PF EM cells has been limited to classical histological analsyis of cell clumps present in PF [6,7]. Not surprisingly, the presence of endometrial cells in PF has been reported to vary between 0 - 59% [8]. Using more objective immunocytochemical methods, some investigators [9] reported that PF contains single epithelial cells, rather than endometrial tissue fragments, in women with patent tubes and that these cells might be of endometrial origin. However, there is no evidence that the EM cell concentration in PF is higher during menstruation than in other phases of the menstrual cycle.

Erythrocytes represent a part of the cell population in PF whichalso contains other free floating cells like macrophages, mesothelial cells, lymphocytes, eosinophil and mast cells. Several studies show that there is an increase in erythrocyte count and consecutively in hemoglobin content in the peritoneal fluid of women with peritoneal endometriosis when compared with controls with a normal pelvis [10]. Hemoglobin overload might have numerous cytotoxic effects in the peritoneal environment [11,12]. Its nonprotein moeity, heme, and its ferrous iron core are known as pro-oxidant and proinflammatory molecules [13] and might be involved in the pathogenesis of endometriosis through several mechanisms including induction of oxidative stress, stimulation of cell adhesion, and cytokine production by macrophages [10]. However, it is not known if the PF concentration of red blood cells and hemoglobin is higher during menstruation than during nonmenstrual phases of the cycle. Endometriosis is associated with a state of subclinical peritoneal inflammation, marked by an increased PF volume, increased PF white blood cell concentration (especially macrophages with increased activation status), and increased inflammatory cytokines, growth factors, and angiogenesis-promoting substances [5,14-16]. Moreover, it has been reported in baboons that subclinical peritoneal inflammation occurs both during menstruation and after intrapelvic injection of endometrium and both the incidence and recurrence of retrograde menstruation is increased in baboons with spontaneous endometriosis when compared to healthy controls [17-19]. However, it is not known if the PF concentration of white blood cells is higher during menstruation than during nonmenstrual phases of the cycle.

The lack of knowledge regarding potential differences in the presence and distribution of PF cell populations during menstruation between women with and without endometriosis is a major obstacle with respect to the validity of the Sampson hypothesis. The aim of our study was to test the hypothesis that menstruation is associated with a higher PF concentration of RBCs, WBCs and EM cells when compared to nonmenstrual phases of the cycle.

## Methods

### Patients and sample collection

Our study protocol was approved by the Institutional Ethical and Review Board of Gasthuisberg University Hospital, KU Leuven for the protection of human subjects. Informed consent was obtained from all patients before entry into this study. A diagnostic laparoscopy for investigation of pelvic pain and/or infertility was performed in 107 reproductive age women (range 22-44 years, demographic data in Table 1). A normal pelvis was observed in 48 women. Endometriosis was found in 59 women and was classified (American Society for Reproductive Medicine, 1997) into minimal (n = 25), mild (n = 20), moderate (n = 6) and severe (n = 8) disease. Endometriosis was confirmed histologically in all patients (n = 59). All 107 patients had patent tubes. Samples of PF were collected during the luteal (n = 46), follicular (n = 38) or menstrual (n = 23) phase of the cycle. The PF was aspirated from the pouch of Douglas before any surgical manipulation was started and immediatelly processed. Special precaution was taken to avoid blood or other fluid (saline, methylene blue dye) contamination. Peritoneal washing was not performed. In all PF samples the colour was noted and divided in the following categories: clear, light yellow, yellow, orange, pink, light red, red, dark red.

**Table 1:** Demographic Characteristics of the Study Population (n = 107)

	Endometriosis (n = 59)	Controls (n = 48)
Age (years, mean ± SD)	31.76 ± 3.71	28.27 ± 3.18
Duration of infertility (years, mean ± SD)	2.67 ± 2.06	2.13 ± 1.12
Primary/secondary infertility [n(%)]	39(66.1)/20(33.9)	30(62.5)/18(37.5)
Concurrent medication [n(%)]	2(3.38)	0
Phase of the menstrual cycle [n(%)]		
Menstrual	18(78.26)	5(21.74)
Nonmenstrual total	38(45.23)	46(54.77)
Follicular	22(26.19)	16(19.04)
Luteal	16(19.04)	30(35.73)
Indication for surgery [n(%)]		
Infertility	58(98.3)	45(93.75)
Pelvic pain	1(1.71)	3(6.25)

In the first 32 patients included in our study, PF cells were used for a detailed cytological analysis using Papanicolau staining and immunocytochemical analysis. These 32 patients included 13 controls and 19 patients with minimal (n = 8), mild (n = 5), moderate (n = 2) or severe (n = 4) endometriosis. In these 32 patients, PF samples were collected during the luteal (n = 20), the follicular (n = 5) or the menstrual (n = 7) phase of the cycle.

### Cell counts

Cell counts (leucocytes, erythrocytes) were determined on a Symex SE 9500 cell counter (Sysmex Co., Kobe, Japan). Leucocyte counts below 0.6 × 10^9^/L were redone by the counting chamber method (Nageotte counting chamber, Marienfeld Laboratory Glassware, Lauda-Königshofen, Germany).

### Immunocytochemistry

PF specimens were centrifuged for 10 minutes at 3000 rpm. The pellet was processed and fixed with CytoRich (Becton Dickinson, Franklin Lakes, NJ. USA) then one thin-layer (Papanicolau stain) was prepared with PrepStain (Becton Dickinson, Franklin Lakes, NJ. USA).

All PF samples underwent both Papanicolau and immunocytochemical staining with monoclonal antibodies against CK 7 (1:100; Dako, Glostrup Denmark), CK 8/18 (1:20; Novocastra, Newcastle upon Tyne, UK), Ber-Ep4 (1:200; Dako, Glostrup Denmark), vimentin (1:100; Dako, Glostrup Denmark), calretinin (1:2000; Swant, Bellinzona, Switzerland) and CD68 (1:10; Kp1, Dako, Glostrup Denmark).

These antibodies were selected since they are commonly used in effusion cytology and they are relevant to identify endometrial epithelial or stromal cells, mesothelial cells and macrophages in PF. Ber-Ep4 is a marker for cells with epithelial (in some cases mesothelial) origin. CD68 is specific for cells from monocyte/macrophage lineage, CK7 and CK8/18 are markers for both endometrial epithelial and mesothelial cells, whereas calretinin and vimentin are markers for both endometrial stromal and mesothelial cells (Tables 2 and 3). Immunocytochemistry was done according to our routinely used protocol with respect to the dilution of the primary antibody, application of the heat-induced epitope retrieval and visualization of the antibody complexes through Envision-HRP with DAB (Dako, Glostrup, Denmark). Slides prepared by the PrepStain method were evaluated by an experienced cytopathologist (MD).

**Table 2:** Cell Counts in Menstrual vs. Nonmenstrual Phase of Cycle

	Menstrual phase (n = 23) mean ± SD	Nonmenstrual phase (n = 84) mean ± SD	P value
Leucocytes × 109/L	3.3 ± 1.1	0.8 ± 0.5	0.03
Granulocytes			
Basophilic × 109/L	0.2 ± 0.08	0.04 ± 0.02	0.002
Eosinophilic × 109/L	0.1 ± 0.09	0.04 ± 0.03	0.09
			
Erythrocytes × 1012/L	0.3 ± 0.2	0.02 ± 0.01	0.006
Haematocrit L/L*	0.03 ± 0.01	0.003 ± 0.02	0.01
Haemoglobin g/dL	0.8 ± 0.7	0.1 ± 0.17	0.01

* calculated values

**Table 3:** Immunocytological markers for different cell populations present in peritoneal fluid

Marker	Endometrial epithelial/glandular cells	Endometrial stromal cells	Mesothelial cells	Macrophages	NK cells	Lymphocytes
**Ber Ep4**	+	-	-	-	-	-
**Vimentin**	-	+	+	-	-	-
**Calretinin**	-	+	+	-	-	-
**Cytokeratin7**	+	-	+	-	-	-
**Cytokeratin8**	+	-	+	-	-	-
**Cytokeratin18**	+	-	+	-	-	-
**CD68**	-	-	-/+	+	-	-

### Statistical analysis

Numerical data were analysed using MS Office Excel (version 6.0; Microsoft Corporation, Redmond, WA, USA). Kolomogorov-Smirnov/Lilliefors and Shapiro-Wilks test was used to test normality to determine whether parametric or non-parametric tests were to be used in further analyses. For statistical analyses, Mann-Whitney and Fisher exact tests were performed. Statistical calculations were performed by using the GraphPad Prism version 5. 00 for Windows, (GraphPad Software, San Diego California USA), and *P *≤ 0.05 was considered significant.

## Results

In comparison with the nonmenstrual phase of the cycle (n = 84), analysis of PF during menstruation (n = 23) showed a 3 fold increased concentration of leucocytes, a 4 fold increase in the concentration of basophilic granulocytes, and a 13, 10 and 8 fold increase in the concentration of erythrocytes, hematocrit and hemoglobin, respectively (Table 2). Morphological evaluation of the Pap-stained specimens showed that the prevalence in PF of single cells with an endometrial phenotype was low and comparable during menstruation (1/7), follicular phase (1/5), and luteal phase (2/20 including one case of single cells and one case of a group of cells). In contrast, mesothelial cells and histiocytes were present in all PF samples.

Mesothelial cells stained positively with CK7, CK8/18, vimentin, calretinin and sometimes weakly with CD68. Cells positive for epithelial marker Ber-Ep4 were not observed, except in two patients investigated during menses who had a few positive cells. In 9/31 patients, the single cell population contained 10-50% cells with very weak, probably nonspecific staining for calretinin. In all patients 50-98% of single cells were strongly positive for both vimentin and CD68. Most of the single cells present in PF were histiocytes or belonged to the monocyte/macrophage lineage. The prevalence of mesothelial cells and macrophages staining positively for the antibodies tested (Table 4) was comparable between menstrual and nonmenstrual phases of the cycle and between patients with and without endometriosis.

**Table 4:** Prevalence of patients with PF cells stained positively by immunocytochemistry for 7 different cell markers among 32 patients with (endo, n = 19) or without (control, n = 13) endometriosis

Marker	Endometrial epithelial/glandular cells		Endometrial stromal cells		Mesothelial cells		Macrophages/Monocytes	
	Endo	Contr	Endo	Contr	Endo	Contr	Endo	Contr
**Ber Ep4**								
Total	2/19	0/13	0/19	0/13	0/19	0/13	0/19	0/13
Menstrual	2/5	0/2	0/5	0/2	0/5	0/2	0/5	0/2
Follicular	0/4	0/1	0/4	0/1	0/4	0/1	0/4	0/1
Luteal	0/10	0/10	0/10	0/10	0/10	0/10	0/10	0/10
**Vimentin**								
Total	0/19	0/13	17/19	11/13	17/19	11/13	0/19	0/13
Menstrual	0/5	0/2	5/5	2/2	5/5	2/2	0/5	0/2
Follicular	0/4	0/1	4/4	1/1	4/4	1/1	0/4	0/1
Luteal	0/10	0/10	8/10	8/10	8/10	8/10	0/10	0/10
**Calretinin**								
Total	0/19	0/13	11/19	9/13	11/19	9/13	0/19	0/13
Menstrual	0/5	0/2	4/5	1/2	4/5	1/2	0/5	0/2
Follicular	0/4	0/1	2/4	1/1	2/4	1/1	0/4	0/1
Luteal	0/10	0/10	5/10	7/13	5/10	7/10	0/10	0/10
**Cytokeratin7**								
Total	10/19	6/13	0/19	0/13	10/19	6/13	0/19	0/13
Menstrual	3/5	2/2	0/5	0/2	3/5	2/2	0/5	0/2
Follicular	3/4	1/1	0/4	0/1	3/4	1/1	0/4	0/1
Luteal	4/10	3/10	0/10	0/10	4/10	3/10	0/10	0/10
**Cytokeratin8**								
Total	4/19	3/13	0/19	0/13	4/19	3/13	0/19	0/13
Menstrual	1/5	0/2	0/5	0/2	1/5	0/2	0/5	0/2
Follicular	1/4	0/1	0/4	0/1	1/4	0/1	0/4	0/1
Luteal	2/10	3/10	0/10	0/10	2/10	3/10	0/10	0/10
**Cytokeratin18**								
Total	4/19	3/13	0/19	0/13	4/19	3/13	0/19	0/13
Menstrual	1/5	0/2	0/5	0/2	1/5	0/2	0/5	0/2
Follicular	1/4	0/1	0/4	0/1	1/4	0/1	0/4	0/1
Luteal	2/10	3/10	0/10	0/10	2/10	3/10	0/10	0/10
**CD68**								
Total	0/19	0/13	0/19	0/13	0/19	0/13	17/19	11/13
Menstrual	0/5	0/2	0/5	0/2	0/5	0/2	5/5	2/2
Follicular	0/4	0/1	0/4	0/1	0/4	0/1	4/4	1/1
Luteal	0/10	0/10	0/10	0/10	0/10	0/10	8/10	8/10

Endo = patients with endometriosis

Contr = patients with a normal pelvis

## Discussion

To the best of our knowledge, our study presents for the first time evidence that menstruation in women is associated with increased PF concentration of leucocytes and erythrocytes when compared to nonmenstrual phases of the cycle. Our data indicate that menstruation is associated with pelvic inflammation and are in agreement with our previous observation in nonhuman primates demonstrating higher levels of white blood cells in PF during menstruation [2].

Our data also show that menstruation is associated with a significant increase in erythrocyte count and consecutively in free hemoglobin content in PF, and provide a quantitative basis for the concept of retrograde menstruation. Hemoglobin accumulation in the peritoneal cavity may be a consequence of increased influx caused by red blood cell degradation, from retrograde menstrual reflux and/or a deficiency in the hemoglobin inactivating system [12] and may be involved in the pathogenesis of endometriosis [10].

In the context of the pathogenesis of endometriosis, retrograde menstruation has to be diagnosed not only as an increased presence of erythrocytes in PF but also as the presence of endometrial cells in PF. Nevertheless, many investigators [20-23] have interpreted the presence of red stained PF at the time of menstruation as sufficient evidence for retrograde menstruation of EM cells. However, it is well known that red-stained PF can be observed not only during menstruation but also during the first 5 days after ovulation [24] and during other phases of the cycle [23,25] and that there is only a weak correlation between the presence of endometrial cells and the color of PF [26].

In our study, we could not confirm the hypothesis that the prevalence and/or quantity of EM cells in PF is increased in women with endometriosis (when compared to controls) and increased during menstruation (when compared to nonmenstrual phases of the cycle). Scientific evidence related to this hypothesis is controversial for several reasons.

Firstly, all studies addressing PF cytology are limited by small sample size, variable and sometimes unspecified phases of the cycle studied, subjective methods to define or detect EM cells or tissue in PF, and prior flushing of uterus and tubes via hysteroscopy before laparoscopic aspiration of PF cells.

Secondly, most investigators have used Papanicolaou or Giemsa staining to detect EM cells in PF and have considered clusters of cells in PF with positive staining to be EM cells [23,26]. However, there is no proof that PF clusters of cells represent EM cells, since they may also include mesothelial cells or macrophages and hystiocytes. Indeed, recent evidence [25] suggests that EM cells rarely agglutinate in PF to become macroscopically visible tissue fragments containing endometrial glands and stroma during menstruation (in only 17% or 3/18 cases and similar in women with (2/9) and women without (1/9) endometriosis) or during all combined phases of the cycle (in only 16% or 16/99 cases and similar in women with (13/65 or 20%) and women without (3-34 or 9%) endometriosis).

Thirdly, immunocytological identification of EM cells in PF is not evident as reported in our study and in 3 papers published by 3 others groups of investigators [9,25,27].

Comparison between these 4 studies is difficult since native PF cells were analyzed in our study and in only one other paper [9] whereas PF cell cultures were used in the 2 other reports [25,27]. It is possible that immunocytological detection of EM cells is facilitated after cell culture when compared to analysis of EM cells in native PF. In our study the PrepStain method was used for the first time to study the cytology of native PF in women with endometriosis. This liquid-based preparation technique is adequate for the immunocytochemical study of numerous cell lines in effusion specimens [28] since cells are transferred directly into the liquid medium at the time of collection and therefore the artefact of air-drying artifact, a culprit in many limited or inadequate conventional smears, is prevented. The results of our study are in line with a previously published immunocytological analysis of PF cells [9] obtained during the early follicular phase from 8 women with endometriosis and 8 with a normal pelvis. In that study [9] all PF samples except one contained cells stained positively with monoclonal antibodies against vimentin, cytokeratin 18 and 19, and 9 out of 16 PF samples contained cells that stained positively with monoclonal antibody BW495/36, an epithelial marker present in endometrium and absent in peritoneal epithelium. These data and our data are consistent with the interpretation that EM cells are present in PF from patients with patent fallopian tubes, and that their immunocytological staining profile is not different between women with and without endometriosis. However, among PF cells obtained during the late follicular phase prior to uterine flushing and cultured in vitro, other investigators could not identify EM cells that were immunocytologically positive for cytokeratins 5/7/8/14/19, cytokeratin 7/18, epithelial marker: BW495/36, HMFG 2, EM epithelial marker NEND-3 or ovarian carcinoma related markers OV-TL3, OV-TL10, OC-125 [27].

Fourthly, it is very difficult to identify with 100% certainty specific PF cell types by specific immunocytological markers, since endometrial epithelial, endometrial stromal, mesothelial cells and macrophages all stain positively for more than one marker (Table 3). For example, it was impossible to make a firm distinction in our study between endometrial stromal cells and mesothelial cells, since both cell types stained positively for vimentin and calretinin. It can be argued that our study was limited by the fact that endometrial stromal cell marker CD10 was not used, but the problem is that also other cell types stain positively for CD10, most importantly cervical stromal cells [29] and other cell types (normal renal tubular and glomerular cells, renal carcinoma, hepatocellular carcinoma lymphoid cells, mesonephric tumors, and acute lymphoblastic leukemia and lymphoma).

Taking together the evidence from our study and other studies, it is remarkable to conclude that even today there is no sound scientific evidence based on the analysis of PF fluid cells that retrograde menstruation is associated with the increased presence of endometrial cells in PF. Recent in vitro evidence demonstrating that attachment in vitro of endometrial cells on mesothelial cells occurs within 1 hour [30] supports the possible explanation that EM cells, refluxed via the Fallopian tube during menstruation into the pelvic cavity, attach in a very short time to the peritoneal wall before they are detectable as free floating cells or clusters in PF. However, it is also possible that endometriosis does not (only) arise from retrograde menstruation, but may also be a consequence of mesothelial metaplasia induced by menstruation or other factors. In support of this metaplasia/induction theory, peritoneal endometriosis lesions appear to contain only stromal endometriosis in 45% of the cases, and stromal endometriosis can be found in about 7% of macroscopically normal peritoneum [31].

## Conclusion

The results of our study demonstrate for the first time that menstruation in women is associated with an increased PF concentration of leucocytes, erythrocytes and hemoglobin when compared to nonmenstrual phases of the cycle, supporting the concept of retrograde menstruation. An increased PF concentration of PF endometrial cells was not observed during menstruation when compared to nonmenstrual phases of the cycle.

## List of abbreviations

AFS: American Fertility Society; CK 7: cytokeratin 7; CK 8/18: cytokeratin 8/18; CD68: cluster of differentiation 68

## Competing interests

The authors declare that they have no competing interests.

## Authors' contributions

AB participated in the design of the study, analysis and interpretation of data, and drafted the manuscript. SD participated in the design of the sudy, data collection, statistical analysis of the data. MD carried out the immunohistological and cytological examinations.

WG carried out the laboratory examination of the peritoneal fluid samples. FV participated in the critical revision of the manuscript for important intellectual content, TD conceived of the study and participated in its design and in the statistical anlysis of the data, coordination and helped to draft the manuscript. All authors read and approved the final manuscript.
